# From Classroom to Operating Room: Addressing Shortcomings in Gender Affirmation Surgical Education

**DOI:** 10.2196/71843

**Published:** 2026-06-04

**Authors:** Samuel G Robinson, Alireza Hamidian Jahromi

**Affiliations:** 1 Lewis Katz School of Medicine, Philadelphia, PA, United States; 2 Division of Plastic and Reconstructive Surgery, Temple University Medical Center, 3401 N Broad St, Philadelphia, PA, 19140, United States, 1 (800) 836-7536

**Keywords:** medical training, education, transgender, residency, plastic surgery

## Abstract

The prevalence of American youth identifying as transgender has doubled in the past five years, emphasizing the crucial role of gender affirmation surgery (GAS) in treating gender dysphoria. However, the current health care infrastructure faces challenges in meeting the escalating demand for GAS interventions. Transgender and gender-diverse patients encounter barriers such as travel and extended waitlists for specialized surgeons. This viewpoint highlights the insufficient exposure to GAS in medical education, spanning from medical students to attending surgeons, and examines the uneven distribution of GAS practitioners across specialties. The absence of formal training and board certification compounds the issue, urging a comprehensive reevaluation of medical education to ensure quality care for the expanding transgender and gender-diverse population.

The percentage of American youth (aged 13‐17 years) identifying as transgender has doubled in the past five years, from 0.7% in 2017 to 1.43% in 2022 [1,2]. With gender affirmation surgery (GAS) serving a vital role in the treatment of gender dysphoria, we must ask if our current infrastructure is prepared to meet the increasing demand for GAS interventions, both in terms of clinical management and for the training of the next generation of gender affirmation–trained surgeons.

It is well-documented that transgender and gender-diverse patients face increased barriers to health care compared to cisgender patients. Patients are often forced to travel out of state to access treatment from a gender affirmation–trained surgeon [3]. Even after locating a gender affirmation–trained surgeon, patients commonly experience prolonged waitlist periods, reflecting broader surgical capacity constraints as well as the limited number of surgeons offering procedure-specific, gender-affirming care [4]. This discrepancy is expected to increase as the transgender and gender-diverse population grows. The medical education system must react to the evolving needs of this already marginalized patient population by working to expand the number of GAS practitioners. Unfortunately, it is the authors’ opinion that the lack of adequate exposure to GAS spans all levels of medical education and training, including medical students, residents, fellows, and attending surgeons from different surgical disciplines.

The current pool of specialties providing gender-affirming surgical care includes plastic surgeons, urologists, otolaryngologists, general surgeons, surgical oncologists, gynecologists, and oral maxillofacial surgeons, with plastic surgeons making up the vast majority of GAS practitioners [4]. It is estimated that a total of 660 physicians in the United States offer at least one GAS procedure [4]. When compared to the United States’ 1.6 million transgender individuals, there is one gender-affirming surgeon per 2400 transgender patients, who may rely on surgical intervention for the treatment of gender dysphoria. Unlike population-based ratios used in other surgical specialties, demand for GAS is concentrated within a subset of patients and limited by procedure specificity, such that population-level ratios likely underestimate unmet surgical need [5-7]. We have to bear in mind that each transgender and gender-diverse individual may be interested in GAS in more than one area of his/her/their body. Additionally, some of the surgical procedures may require multiple surgical steps. For instance, masculinization bottom surgery may involve metoidioplasty with or without urethral lengthening, suprapubic catheter placement, hysterectomy, oophorectomy, phalloplasty, glansplasty, scrotoplasty, monsplasty, and the insertion of testicular and/or penile implants. To make matters worse, the geographic distribution of plastic surgeons and transgender and gender-diverse individuals do not align. Plastic surgeons are more often located in zip codes with higher household incomes, while transgender and gender-diverse people are more commonly located in areas with lower household incomes [8,9]. Further identifying surgeons with adequate experience in GAS is extremely difficult, especially for patients with lower health literacy. While not currently in development, the formation of a board-certifying body in GAS could provide both practitioners and patients the tools needed to increase access to care. Board certification is associated with improved patient outcomes [10] and would serve as a means for transgender and gender-diverse patients to evaluate whether a surgeon or procedure is a safe and smart option. There remains no formal board certification for surgeons who practice GAS. This is in part due to the lack of formal training available for surgeons interested in the specialty.

A cross-sectional study from 2022 identified only 7 GAS fellowships available for plastic surgeons in the United States [11]. Each fellowship lasts 12 months and offers an inconsistent mix of training in feminizing or masculinizing chest and genital surgeries and facial feminization, with some only offering one of the three surgical categories. The presence of designated GAS fellowships reflects the increasing demand for gender affirmation–trained surgeons. However, with 525 plastic surgeons offering GAS procedures in the United States [4], it is safe to assume the vast majority of contemporary gender-affirming surgeons are not fellowship trained. This fact alone leads one to question if residency programs are offering sufficient education on the subject to warrant practitioners the means to include GAS in their practice.

In contrast to the United States, where no formal certification or credentialing pathway is required for surgeons to perform GAS, several countries have national or regional approval mechanisms that regulate who is permitted to provide GAS. In the United Kingdom, surgeons must be credentialed through National Health Service (NHS)–commissioned gender services and operate within designated centers [12]. These models function as de facto training and certification pathways. However, by limiting GAS to a small number of designated centers, these centralized systems may constrain surgical capacity and geographic access. Hybrid approaches to education, such as expanding structured GAS training within US residency programs, may better balance quality and availability, although a direct international comparison remains challenging.

The Accreditation Council of Graduate Medical Education (ACGME) designates the education components required for a plastic surgery residency program to be accredited. A resident must complete at least 1150 surgical cases in order to graduate [13]. While many of the required cases involve surgical technique similar to those used in GAS procedures, none of the 1150 cases specifically refer to GAS interventions. For most surgeons performing GAS today, the unregulated exposure they have received in residency is the extent of their training in this specialty. ACGME is encouraged to expand their required plastic surgery resident competencies to include GAS-specific operations so that the growing transgender and gender-diverse patient population receives quality care even when a practitioner is not trained in a GAS fellowship. Importantly, high-quality exposure to gender-affirming surgery during residency does not require fellowship-trained faculty, but rather structured operative involvement, multidisciplinary care, and longitudinal patient follow-up [14,15]. With this said, the issue of inadequate education on the topic originates far before residency training begins.

The Liaison Committee on Medical Education (LCME) requires all MD-accredited medical schools to include curricular content on health care disparities including gender identity [16]. Despite this requirement, only 26.1% of the 4070 surveyed medical students feel comfortable discussing GAS with transgender and gender-diverse patients [17]. To what extent gender-diverse care is included in today’s medical school curriculum is largely unknown. Medical schools vary significantly in the manner in which they mandate or voluntarily offer gender-based education, with some requiring students to attend multiple lecture series on the topic and some simply allowing students to seek out the knowledge if they please. A survey encompassing students from 175 medical schools found that 37% of respondents attended institutions where mandatory preclinical instruction was provided on “LGBT patient care” [17]. With medical schools choosing to disregard the importance of gender-conscious care in their curriculum, the transgender and gender-diverse patient population relies on students seeking out the knowledge on their own. The medical education system originates in the preclinical classrooms of medical schools, where students will carry forward the principles they are taught into their careers as resident physicians, clinical fellows, and eventually attendings. The educational system’s disregard for gender-focused learning in medical school is materializing as consequences shouldered by transgender and gender-diverse patients. This marginalized population is desperately seeking out gender-affirming surgeons who do not have the time, resources, or formal training needed to meet the needs of the growing transgender and gender-diverse community.

## Abbreviations

ACGME: Accreditation Council of Graduate Medical Education
GAS: gender affirmation surgery
LCME: Liaison Committee on Medical Education
NHS: National Health Service
